# Cloning and tissue distribution of novel splice variants of the ovine ghrelin gene

**DOI:** 10.1186/s12917-014-0211-x

**Published:** 2014-09-06

**Authors:** Moira Menzies, Inge Seim, Peter Josh, Shivashankar H Nagaraj, Michael Lees, Carina Walpole, Lisa K Chopin, Michelle Colgrave, Aaron Ingham

**Affiliations:** CSIRO Animal, Food and Health Sciences, St Lucia, QLD Australia; Ghrelin Research Group, Translational Research Institute, Institute of Health & Biomedical Innovation and APCRC-Q, Queensland University of Technology, 37 Kent St., Woolloongabba, Brisbane, QLD Australia; Queensland Centre for Medical Genomics, Institute for Molecular Bioscience, the University of Queensland, St Lucia, QLD Australia

## Abstract

**Background:**

The ghrelin axis is involved in the regulation of metabolism, energy balance, and the immune, cardiovascular and reproductive systems. The manipulation of this axis has potential for improving economically valuable traits in production animals, and polymorphisms in the ghrelin (*GHRL*) and ghrelin receptor (*GHSR*) genes have been associated with growth and carcass traits. Here we investigate the structure and expression of the ghrelin gene (*GHRL*) in sheep, *Ovis aries.*

**Results:**

We identify two ghrelin mRNA isoforms, which we have designated Δex2 preproghrelin and Δex2,3 preproghrelin. Expression of Δex2,3 preproghrelin is likely to be restricted to ruminants, and would encode truncated ghrelin and a novel C-terminal peptide. Both Δex2 preproghrelin and canonical preproghrelin mRNA isoforms were expressed in a range of tissues. Expression of the Δex2,3 preproghrelin isoform, however, was restricted to white blood cells (WBC; where the wild-type preproghrelin isoform is not co-expressed), and gastrointestinal tissues. Expression of Δex2 preproghrelin and Δex2,3 preproghrelin mRNA was elevated in white blood cells in response to parasitic worm (helminth) infection in genetically susceptible sheep, but not in resistant sheep.

**Conclusions:**

The restricted expression of the novel preproghrelin variants and their distinct WBC expression pattern during parasite infection may indicate a novel link between the ghrelin axis and metabolic and immune function in ruminants.

## Background

The peptide hormone ghrelin has a wide range of physiological functions, including a role in appetite stimulation, and energy balance and regulation of the immune and reproductive systems [1–4]. Ghrelin is most abundantly expressed in the stomach of monogastrics and the abomasum (glandular stomach) of ruminants, and it is also expressed in a wide range of other tissues [3,5–7]. In order to bind and activate its cognate receptor, the growth hormone secretagogue receptor 1a (GHSR1a), ghrelin is post-translationally octanoylated by the enzyme ghrelin-*O*-acyl transferase (GOAT) [8,9].

The ghrelin axis (which includes ghrelin, GHSR, and GOAT) is a potential target for improving production in ruminant species through the manipulation of feeding, growth, body composition and immune and reproductive functions [10–12]. Ghrelin or GHSR polymorphisms have been associated with enhanced food intake, growth and body conformation in cattle [13,14], and GHSR polymorphisms have been associated with carcass traits in sheep [10]. GHSR mRNA expression was elevated in the pituitary gland of a line of sheep selected for increased fat cover, compared to a lean sheep selection line [15]. Ghrelin and GHSR1a are thought to regulate reproduction in many species and are expressed in reproductive tissues of sheep [16–18].

We previously reported differential ghrelin expression in the gut (mRNA) and blood (peptide) of sheep selected for gastrointestinal nematode resistance compared to susceptible sheep [19], suggesting that ghrelin may play a role in the immunity to parasite infection. Here, we report a detailed characterization of the ghrelin gene (*GHRL*) in the sheep, *Ovis aries*, and the discovery of novel ghrelin variants with distinct mRNA expression in white blood cells in response to helminth infection.

## Results and discussion

### Ghrelin gene structure

**Figure 1:**
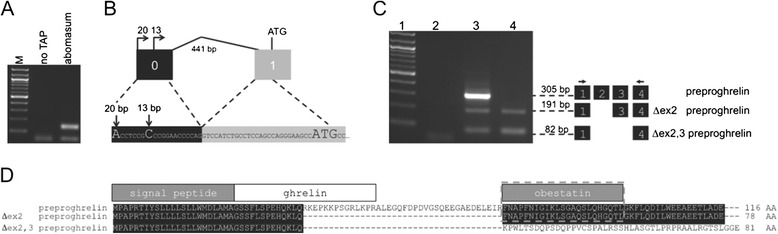
**Characterization of the sheep ghrelin gene. (A)** Identification of the ovine *GHRL* transcription start sites. 5′ RLM-RACE of abomasum. M = NEB molecular weight marker 100; no TAP = negative TAP control. **(B)** Partial transcript sequence showing exon 0 and exon 1. *GHRL* exon 0 is shown as a black box, exon 1 as a grey box, introns as horizontal lines. Transcription start sites in exon 0 are capitalized. Size (bp) are shown above each exon. The translation initiation site of preproghrelin is shown as ATG. **(C)** Ethidium bromide stained agarose gel showing the expression of ghrelin (305 bp), exon 2-deleted preproghrelin (Δex2 preproghrelin, 191 bp) and exon 2,3-deleted preproghrelin (Δex2,3 preproghrelin, 82 bp) amplified from the abomasum (Lane 3) and white blood cells (Lane 4) of sheep. The exon structure corresponding to each RT-PCR product is depicted by the adjacent boxes, and the positions of RT-PCR primers are indicated by arrows above exons. Lane 1 contains a 100 bp molecular weight marker and Lane 2 the no template control. **(D)** Predicted translation and alignment of sheep preproghrelin variants. The signal peptide, ghrelin and obestatin are shown as boxes above corresponding coding sequences. Wild-type preproghrelin code for a 27 amino acid (AA) ghrelin peptide, while Δex2 preproghrelin and Δex2,3 preproghrelin code for a truncated 13 AA ghrelin peptide. Obestatin peptide (sequence indicated by a dotted line) is encoded by the C-terminus of the wild-type preproghrelin and Δex2 preproghrelin, whereas Δex2,3 preproghrelin has a unique 45 amino acid carboxyl terminal sequence.

Partial structure and sequence of the ghrelin gene (*GHRL*) has been determined in a number of ruminants including cattle [20], sheep [21], goat [22] and water buffalo [23], however, no complete gene structure exists for the sheep gene. To investigate ghrelin gene structure and expression in the sheep, 5′ RLM-RACE and RT-PCR (using primers spanning the canonical exons 1 to 4 of preproghrelin) were performed using abomasum (glandular stomach) and white blood cells (WBC). For consistency, we follow the exon numbering nomenclature of the human and murine *GHRL* genes [24,25]. Sequence analysis of 5′ RLM-RACE products revealed two alternative transcriptional start sites (TSS) present in a short 5′ untranslated exon, 13–20 bp in length, which was previously termed exon 0 (Figure 1A and B). These TSS were found in both the abomasum and WBC.

Using RT-PCR we demonstrated that the canonical preproghrelin variant (305 bp) is expressed in the abomasum and white blood cells (Figure 1C). Sequencing confirmed that this 305 bp RT-PCR product consisted of 4 exons and the corresponding full-length transcript would encode a 116 amino acid preproghrelin protein, identical to the sheep reference sequence [GenBank: NM_001009721]. The canonical coding exons of the ovine ghrelin gene are exons 1 (135 nt), 2 (114 nt), 3 (109 nt) and 4 (~150 nt). Although the predominant form of preproghrelin is 117 amino acids (AA) in length and mature ghrelin is 28 AA in most mammals, the ruminant form of preproghrelin is 116 AA and ghrelin is a 27 AA peptide, resulting from alternative splicing which removes a codon for glutamine [20,22,26–28].

**Figure 2:**
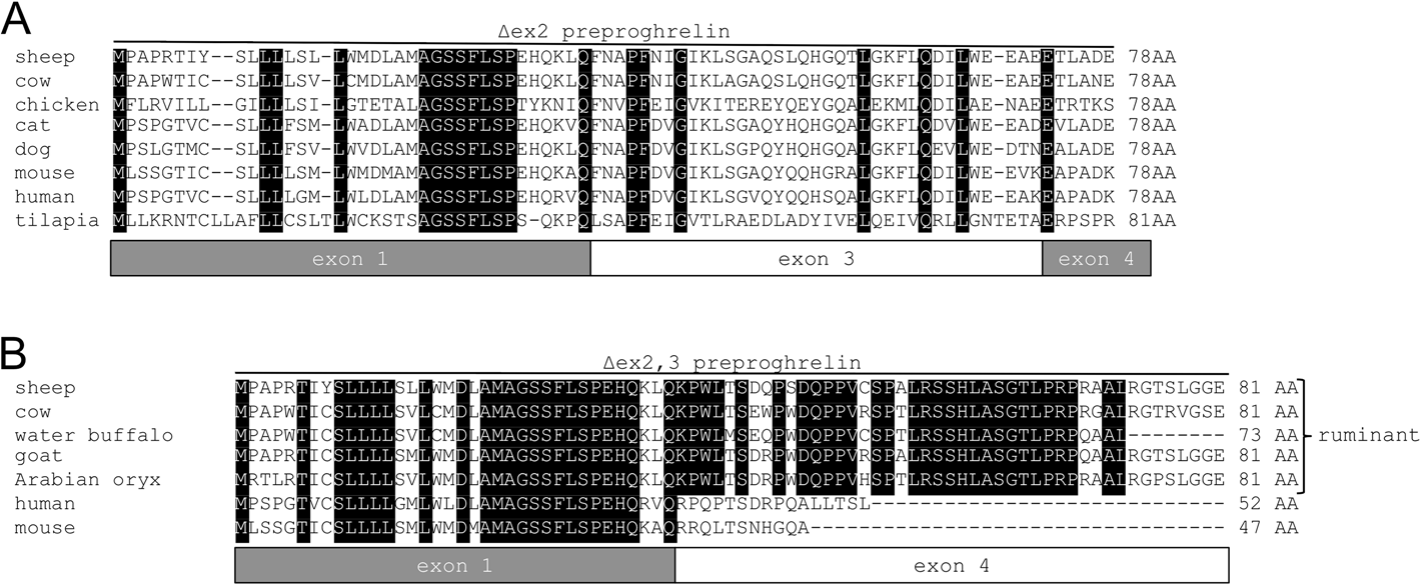
**Alignment of** Δ**ex2,3 preproghrelin and** Δ**ex2,3 preproghrelin.** Alignment of **(A)** Δex2,3 preproghrelin and **(B)** Δex2,3 preproghrelin peptide sequences. The exonic structure containing the sequence coding for the peptide is shown below the sequence. Downstream exon 4 sequence data was not available for the water buffalo and Δex2,3 preproghrelin for this species is shown as 73 amino acids.

Two additional RT-PCR products, 191 bp and 82 bp in size, were also amplified (Figure 1C). Sequencing revealed that the 191 bp mRNA isoform [GenBank: JQ655468] lacks exon 2 and has been designated Δex2 preproghrelin. Translation of Δex2 preproghrelin would produce a 78 amino acid peptide, consisting of the preproghrelin signal peptide, followed by a 55 amino acid peptide that includes a C-terminally truncated ghrelin peptide (13 amino acids) and the obestatin sequence (which is encoded by exon 3) (Figure 1D). Interestingly, although exon 2 is absent from the Δex2 preproghrelin mRNA sequence, the open reading frame is conserved in mammals (Figure 2A). Obestatin, a peptide hormone derived from the ghrelin preprohormone [29], has independent functions from its sibling peptide, including autocrine/paracrine roles in the pancreas and adipose tissue [30].

The 82 bp amplicon [GenBank: JQ655469] lack exons 2 and 3 and has been designated Δex2,3 preproghrelin. Like exon 2-deleted preproghrelin, it is predicted that translation of this variant would produce an 81 amino acid peptide that contains the preproghrelin signal peptide, followed by the first 13 amino acids of the mature ghrelin peptide (Figure 1D). A unique 45 amino acid C-terminal peptide (lacking obestatin) is predicted to follow, as removal of the non-symmetrical exon 3 creates a frame shift. In contrast to Δex2 preproghrelin which is conserved in mammals, only ruminants of the Bovidae family harbor an intact Δex2,3 preproghrelin open reading frame (Figure 2B). This include sheep, goat, cow, the water buffalo (*Bubalus bubalis*), and the antelope Arabian oryx (*Oryx leucoryx*). The 45 amino acid peptide sequence of Δex2,3 preproghrelin does not present any known sequence motifs that could indicate its possible function (data not shown).

### Expression of preproghrelin variants and the ghrelin acylation enzyme in ovine tissues

The tissue distribution of ghrelin in sheep has been reported previously [21]. In the earlier study, RT-PCR primers spanned exons 2 and 4 of the ghrelin gene, excluding the possible amplification of the Δex2 preproghrelin and Δex2,3 preproghrelin variants. We screened 20 different cell and tissue types for wild-type preproghrelin transcript, the two novel preproghrelin variants and the ghrelin acylation enzyme GOAT (encoded by *MBOAT4*) using RT-PCR. We found that the expression of wild-type preproghrelin and the novel splice variants was tissue specific (Table 1). Full-length preproghrelin was expressed in all tissues examined, with the exception of the urinary bladder, skeletal muscle and WBC (Table 1). Δex2 preproghrelin expression was also detected in many tissues, including the gastrointestinal tract (abomasum, small intestine, colon), lymphoid tissue (lymph nodes and Peyer’s patches), lung, WBC, and urinary bladder (Table 1). Interestingly, the expression of Δex2,3 preproghrelin was restricted to WBC, abomasum, small intestine and lymphoid tissue associated with the small intestine. The enzyme GOAT, (encoded by the gene *MBOAT4*) catalyses the octanoylation of ghrelin, allowing it to signal through its cognate receptor, GHSR1a [8,9]. GOAT is expressed in the abomasum (glandular stomach) and pancreas in sheep, and expression has been demonstrated in equivalent tissues in other species [8,9,31]. GOAT expression was not detected in a number of sheep tissues that expressed ghrelin variants, including white blood cells, lung and venous tissue (Table 1), suggesting that the ghrelin peptide or ghrelin peptide variants expressed in these tissues are not acylated. The unmodified form of ghrelin, desacyl ghrelin (or des-ghrelin), is also functional, but acts through an unidentified alternative ghrelin receptor in some cell and tissues [32].

**Table 1:** **Tissue distribution of ‘wild-type’ preproghrelin, the** Δ**ex2 preproghrelin and** Δ**ex2,3 preproghrelin variants (encoded by** ***GHRL*** **), and GOAT (encoded by** ***MBOAT4*** **) in sheep, determined using qualitative RT-PCR**

**Tissue**	**Preproghrelin**	Δ**ex2 preproghrelin**	Δ**ex2,3 preproghrelin**	**GOAT**
Aorta	+	-	-	-
Abomasum	+	+	+	+
Bladder	-	+	-	-
Colon	+	+	-	+
Heart muscle	+	-	-	+
Hypothalamus	+	+	-	-
Kidney	+	-	-	+
Large intestine	+	+	-	+
Liver	+	+	-	+
Lung	+	+	-	+
MLN	+	+	-	+
Pancreas	+	+	-	+
Salivary gland	+	-	-	+
Skeletal muscle	-	-	-	+
Small intestine	+	+	+	+
Spleen	+	-	-	+
Trachea	+	-	-	-
Tooth	+	+	-	+
Vein	+	+	-	-
White blood cells	-	+	+	-

MLN denotes Mesenteric Lymph Node. + = expressed. - = not detected.

### The Δex2 preproghrelin and Δex2,3 preproghrelin variants are responsive to parasite infection

**Figure 3:**
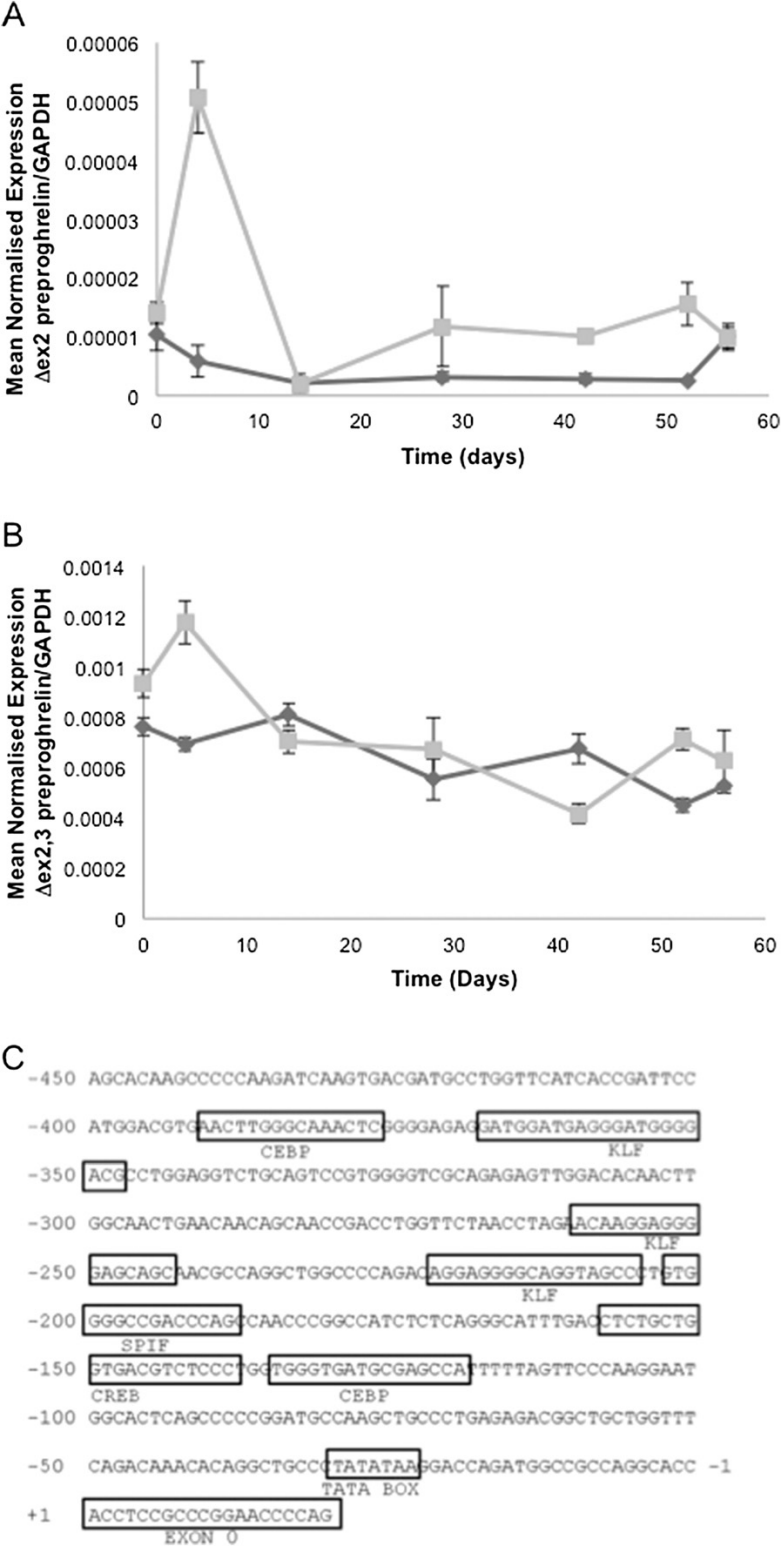
**Expression of ghrelin variants in white blood cells of sheep and overview of the proximal promoter region of the ovine ghrelin gene.** Expression of ghrelin variant transcripts, Δex2 preproghrelin **(A)** and Δex2,3 preproghrelin **(B)** in white blood cells from genetically resistant (dark grey diamond symbol) or susceptible (light grey square symbol) sheep, following infection with *Haemonchus contortus,* demonstrated using real time RT-PCR. Results at each time point indicate mean and standard error from a group of 8 sheep. **(C)** Promoter region of the ovine ghrelin gene (*GHRL*). Predicted transcription factor binding sites are boxed and the transcription factor families that target this sequence are shown below the sequence. The transcription factor sites identified are also conserved in the promoter region of bovine ghrelin. The TATA box, a consensus sequence upstream of the transcription start site that plays a role in transcription initiation, and the first exon of the ghrelin gene (exon 0) are also shown.

We noted that white blood cells expressed Δex2 preproghrelin and Δex2,3 preproghrelin, but not wild-type preproghrelin transcripts (Figure 1C and Table 1). On the basis of these findings, we hypothesized that preproghrelin variants may be differentially expressed in response to parasitic worm (helminth) infection. Blood samples were generated in an earlier experiment in which sheep from the parasite resistant and susceptible lines of a selection flock were challenged with the highly pathogenic nematode, *Haemonchus contortus* [33]. Expression of Δex2 preproghrelin and Δex2,3 preproghrelin was determined in white blood cells (WBC) at a number of time points after infection (Figure 3A and B). While the wild-type preproghrelin was not amplified in any WBC samples (data not shown), expression of both novel preproghrelin variants increased in WBC in susceptible sheep 4 days after infection. From day 10, expression of the variants remained constant.

Having established that the novel Δex2 preproghrelin and Δex2,3 preproghrelin variants display a distinct expression pattern in white blood cells following parasite infection, we interrogated the proximal promoter region of the sheep ghrelin gene. As outlined above, RLM-RACE (Figure 1A and B) revealed that WBCs harbor a transcriptional start site in a short untranslated exon 0. The sequence region upstream of exon 0 of ghrelin was analysed for possible promoter features and transcription factor binding sites. A candidate promoter region, which is conserved between sheep and cattle, was identified in the first 450 bp upstream of exon 0 (Figure 3C). A TATA box likely to provide an RNA polymerase binding site for *GHRL* transcription initiation was present 25 bp upstream of exon 0. Putative binding sites for members of the Ccaat/Enhancer Binding Protein (CEBP), cAMP-responsive element binding proteins (CREB), Krüppel-like factor (KLF) and specificity factor (SP1F) transcription factor families were also identified in this region. Three putative KLF-binding sites are present in the promoter region within 450 base pairs of the *GHRL* transcriptional start site in sheep. KLF4 is highly expressed in the stomach and other cells and tissues, has a similar expression pattern to ghrelin and regulates human ghrelin gene expression in the human AGS stomach cell line [34]. The zinc finger transcription factor KLF4 interacts with CREB and plays a wide range of roles, including the regulation of cell growth and differentiation [34]. Interestingly, evidence is emerging that KLF4 is involved in the pathophysiology of inflammatory diseases, including parasite infection [35–38]. We speculate that KLF4 plays a role in regulating ghrelin expression in response to helminth infection.

### Variations on a theme: alternative splicing of the ghrelin gene

A number of peptide preprohormones and neuropeptides are alternatively spliced to produce different isoforms, or peptides with different functions. It is now appreciated that the ghrelin gene locus is a polyhormone locus that, via alternative splicing and post-translational processing, produces a number of bioactive peptides [25,32]. The peptide hormone, obestatin, is derived from the ghrelin preprohormone [29] and has functions which are independent of ghrelin [30]. Other novel peptides derived from isoforms of the ghrelin preprohormone may also be functional [24,39–42].

We here describe two novel splice variants derived from the sheep ghrelin gene. Both Δex2 preproghrelin and Δex2,3 preproghrelin contain the conserved ghrelin GSSFL amino acid motif, which is the minimum sequence required for binding and stimulation of the ghrelin receptor GHSR1a *in vitro* [43] and for acylation of ghrelin by GOAT [44]. Although the truncated ghrelin variants are likely to have shorter half-lives than full-length ghrelin [45], these isoforms could have both endocrine effects (such as appetite stimulation), as well as autocrine/paracrine actions in peripheral tissues.

A previous study conducted in our laboratory showed differential levels of ghrelin in the gut (mRNA) and blood (peptide) of sheep selected for gastrointestinal helminth resistance compared to susceptible sheep [19], suggesting that ghrelin is associated with immunity to parasite infection. Given a lack of wild-type preproghrelin and differential expression pattern in white blood cell noted between the resistant and susceptible sheep in the current study, these new variants are likely to play a role in immunity. In particular, the finding that the expression of the Δex2,3 preproghrelin variant is limited to white blood cells and the gut, suggests that there is a direct link between tissues of the digestive tract and circulating immune cells. Δex2,3 preproghrelin would be translated into a peptide that contains the minimal active region of ghrelin, and a novel 45 amino acid peptide.

## Conclusions

Although further larger studies are required, we speculate that Δex2 preproghrelin and Δex2,3 preproghrelin are translated into bioactive peptides that function during the early stage of an effective response to gastrointestinal nematodes. In addition, the variants and their derived peptides may be useful in predicting susceptibility and resistance to parasitic infection. The recognition of novel preproghrelin variants should aid in interpreting studies linking expression of the ghrelin gene to phenotypic differences in ruminant production animals.

## Methods

### Animals

RNA samples from white blood cells and abomasum from Merino sheep challenged with *Haemonchus contortus* infection were generated for a previously reported study [33], as described below. This RNA was used for cloning of ghrelin variants and for characterization of ghrelin gene structure. All related procedures were approved by the F. D. McMaster Animal Ethics Committee, CSIRO Livestock Industries (AEC Approval No. 05/58). A range of tissues were collected from three euthanized Merino sheep in accordance with the Australian Code of Practice for the Care and Use of animals for scientific purposes and Innovation Animal Ethics Committee (SA 2009/09/297) and used to determine the tissue expression of the ghrelin variants. Tissues were collected on ice, rapidly frozen using liquid nitrogen and stored at −80°C.

### RNA extraction and reverse transcription

For each tissue sample, 50 mg frozen tissue was wrapped in aluminum foil and immediately transferred to liquid nitrogen. For RNA extraction, the tissue was pulverized on a cold block using a hammer, scraped into RLT buffer (QIAGEN, Hilden, Germany) and immediately homogenized for 2 × 60 s with 0.5 mm glass beads using a Mini-Beadbeater (Biospec Products, Bartlesville, OK). Total RNA was isolated from the homogenized tissues using an RNeasy Plus Mini kit (QIAGEN), with genomic DNA eliminator spin columns, according to the manufacturer’s instructions. Total RNA (2.5 μg) was subjected to a further *DNase I* digestion (amplification grade, Invitrogen, Carlsbad, CA) to ensure complete removal of genomic contamination. RNA 260:280 nm absorbance ratios were 1.9-2.0 and 260:230 nm ratios were between 2.0 and 2.2. For qualitative and quantitative RT-PCRs, cDNA was generated from 2 μg total RNA by reverse transcription using oligo(dT)_20_ primers and 200 units SuperScript III (Invitrogen) in a final volume of 20 μL using a PTC-200 thermal cycler (MJ Research, Watertown, MA), according to the manufacturer’s instructions. The resulting single-stranded cDNA was treated with ribonuclease H (Invitrogen).

### Identification of transcription start sites of the sheep ghrelin gene

5′ rapid amplification of cDNA ends (RACE) was undertaken using the FirstChoice RNA ligase mediated (RLM)-RACE kit (Invitrogen) according to the manufacturer’s instructions. Briefly, 20 μg total RNA was treated with 1.5 U shrimp alkaline phosphatase (Fermentas, Glen Burnie, MD) in a 25 μl total reaction volume. Next, 10 μL phosphatase treated RNA was treated with 5 U tobacco acid pyrophosphatase (TAP, EPICENTRE Biotechnologies, Madison, WI) in a 50 μL reaction to uncap intact RNA. The remaining 10 μL was employed in a no-TAP control reaction, where TAP was substituted with water. RNA was purified using an RNeasy Mini cleanup kit (QIAGEN) and eluted in 12 μL RNase-free water. Next, 0.3 μg/μl of 5′ RACE adapter (Ambion) was ligated to the eluted RNAs in a total volume of 30 μl using 30 U T4 RNA ligase (New England Biolabs, Ipswich, MA). Following a 16 h incubation at 17°C in a PTC-200 thermal cycler (MJ Research), the adapter-ligated RNA was purified (as before) and eluted in 10 μl. Adapter-ligated total RNA (2 μg) from the abomasum and white blood cells was reverse transcribed with 200 units SuperScript III (Invitrogen) using oligo(dT)_20_ primers in a final volume of 20 μl, according to the manufacturer’s instructions. All enzymatic (SAP, TAP, reverse transcription) reactions contained 40U RNasin Plus ribonuclease inhibitor (Promega, Fitchburg, WI). Following reverse transcription, the RACE-ready cDNA was treated with 2U of RNase H (Invitrogen) according to the manufacturer’s instructions.

The first round of RT-PCR was performed with an adapter-specific forward primer and a *GHRL-*specific reverse primer (5OF/R, Table 2). The RT-PCR product (1 μL) was diluted 1/10 in water and then used in a secondary, nested RT-PCR (5IF/R, Table 2). RT-PCRs were performed in a total reaction volume of 50 μL using 1 U Platinum Taq Polymerase (Invitrogen), according to the manufacturer’s instructions. RT-PCR products were purified using a PureLink PCR Purification Kit (Invitrogen), cloned into *pTargeT* (Promega), and transformed into TOP10 chemically-competent cells (Invitrogen). Sequencing reactions were performed using a BigDye Terminator Cycle Sequencing Kit v3 (Applied Biosystems, Foster City, CA, USA) on an ABI PRISM 3100-Avant Genetic Analyzer (Applied Biosystems), after pre-sequencing clean up of excess dye terminator with a CleanSEQ Sequencing Reaction Cleanup Kit (Agencourt Bioscience Corporation, Beverly, MA, USA).

**Table 2:** **Primer sequences used for qualitative and quantitative (q) real time RT-PCR, and RACE and the expected product size**

**Primer name**	**Assay**	**Sequence (5′-3′)**	**Exon position**	**Target gene**	**Amplicon sizes (bp)**
GHRL-1 F	RT-PCR	TTTCTGAGCCCTGAACATCAG	1	*GHRL*	305 canonical (wild-type) preproghrelin
GHRL-4R		GAGAACAGACAGGTGGTTGG	4		
GHRL-5OF	RACE	ATGAATGAACACTGCGTTTGCT	N/A	*GHRL*	174
GHRL-5OR		CAGTTTCTGATGTTCAGGGCTC	1		
GHRL-5IF	RACE	GAACACTGCGTTTGCTGGCT	N/A	*GHRL*	130
GHRL-5IR		GGCCAAGTCCATCCAGAGCA	1		
GHRL-2F	qRT-PCR	CTAAGAAGCCGTCAGGCAGACT	2	*GHRL* (preproghrelin)	153
GHRL-2R		GGGACTGAGCCCCTGACA	3		
GHRL1_3F	qRT-PCR	CAGAAACTGCAGTTCAATGC	1 / 3	*GHRL* (Δex2 preproghrelin)	224
GHRL-4R		CACGTGGTCTCGGAAGTGT	4		
GHRL1_4F	qRT-PCR	CTGCAGAAACCCTGGCTGA	1 / 4	*GHRL* (Δex2,3 preproghrelin)	109
GHRL-4R		CACGTGGTCTCGGAAGTGT	4		
GOAT-mF	qRT-PCR	GGTTTCAAGCTCGAGTTGAAGG	N/A	*MBOAT4* (GOAT)	212
GOAT-mR		AGTAGGTGAGTTTGAAGAGCCC			
GAPDH-F	qRT-PCR	CCTGGAGAAACCTGCCAAGT	N/A	*GAPDH*	209
GAPDH-R		GCCAAATTCATTGTCGTACCA			

### *In silico* promoter analysis

The promoter region of the ovine ghrelin gene was annotated using the Genomatix (Munich, Germany) suite of programs. As the sheep genome sequence had yet to be released, the region upstream of exon 0 of the bovine *GHRL* gene was used for initial development of a promoter model. The MatInspector program was then used to identify possible transcription factor binding sites within each model. Stringent criteria were used in this stage of analysis with a minimum core similarity of 1 and matrix similarity of 0.9 selected. The bovine region was then aligned to the region upstream of the ovine *GHRL* gene transcriptional start site. Regions conserved between the bovine and ovine sequences were conserved candidate promoter elements.

### Non-quantitative expression of preproghrelin variants and the ghrelin acylation enzyme GOAT

RT-PCR primers (Table 2) were designed to examine the splicing pattern of preproghrelin mRNAs (encoded by *GHRL*), as well as the expression of the ghrelin acylation enzyme GOAT (encoded by *MBOAT4*). Preproghrelin variants were amplified using primers in the terminal coding exons (exons 1 and 4) of the canonical (wild-type) preproghrelin coding sequence. RT-PCRs were performed using 200 ng cDNA, and 1 U Platinum *Taq* HIFI Polymerase (Invitrogen) in a final volume of 50 μL using a PTC-200 thermal cycler (MJ Research), according to the manufacturer’s instructions. RT-PCR products were separated by electrophoresis on a 2% agarose gel in Tris-acetate-EDTA (TAE) buffer, stained with ethidium bromide and visualised using ultraviolet light.

RT-PCR products were purified using a PureLink PCR Purification Kit (Invitrogen), or a MinElute PCR Purification kit (QIAGEN), cloned into *pTargeT* (Promega), and transformed into TOP10 chemically-competent cells (Invitrogen). Sequencing reactions were as outlined above.

### Bioinformatic alignment of ghrelin peptide variants

Preproghrelin exon sequences were obtained by BLAST alignments [46] against nucleotide sequences available in the NCBI GenBank database. The recently released draft genome sequence (oryxL1) of the Arabian oryx (*Oryx leucoryx*) was obtained [47] and interrogated using a local instance of BLASTn available in v2.2.26+ of the BLAST + suite [48]. We acknowledge the efforts of the Weill Cornell Medical College in Qatar (WCMCQ) Genomics Core, the Biotechnology Centre of the Ministry of Environment (Qatar) and Wabra Wildlife Preservation (Qatar) in generating the oryx genome sequence. The obtained nucleotide sequences were converted to protein sequences using the ExPASy Translate tool [49] and aligned using MUSCLE [50].

### Quantitative real time reverse transcription (RT-)PCR

Real time RT-PCR assays were designed to quantify expression of canonical ovine preproghrelin transcript (GHRE-2 F/2R), Δex2 preproghrelin (GHRL1_3F/4R) and Δex2,3 preproghrelin (GHRL1_4F/4R) in white blood cells (WBC) following infection with the parasitic helminth *Haemonchus contortus* (Table 2). Real time RT-PCRs for the housekeeping gene, glyceraldehyde 3-phosphate dehydrogenase (*GAPDH*), were performed using the GAPDH-F/R primer pair, as previously described [51].

Briefly, white blood cells (WBC) were collected from Merino sheep exposed to a gastrointestinal helminth challenge with *H. contortus,* as outlined in a previous study [33]. Groups of 8 sheep bred for parasite resistance and 8 sheep bred for parasite susceptibility were individually dosed with a bolus of 5,000 *H. contortus* L3 infectious larvae (Kirby strain). Blood was collected immediately prior to infection (day 0) and on days 4, 14, 28 and 42 post-infection. White blood cells were isolated from each blood sample, RNA extracted and cDNA synthesized, as described above.

Real time RT-PCR was performed using the ABI Prism 7900HT sequence detection system (Applied Biosystems). Each reaction contained 1 × SYBR Green Master Mix (Applied Biosystems), 900 nM each primer and a constant amount of cDNA (corresponding to 10 ng of reverse transcribed RNA for each sample). Three technical replicates were included for each candidate gene. For each of the biological samples, gene expression was quantified by normalizing each target gene against the expression of the reference gene, *GAPDH*, using the Q-GENE statistical analysis package [52]. Q-GENE calculates a Mean Normalised Expression (MNE) ± Standard Error (SE), correcting for amplification efficiencies.
